# Application of the Random Amplified Polymorphic DNA (RAPD) Fingerprinting to Analyze Genetic Variation in Community Associated-Methicillin Resistant *Staphylococcus Aureus* (CA-MRSA) Isolates in Iran

**DOI:** 10.5539/gjhs.v8n8p185

**Published:** 2015-12-17

**Authors:** Sina Mobasherizadeh, Hasan Shojaei, Seyed Asghar Havaei, Kamyar Mostafavizadeh, Fazollah Davoodabadi, Farzin Khorvash, Behrooz Ataei, Abbas Daei-Naser

**Affiliations:** 1 Nosocomial Infection Research Center, Isfahan University of Medical Sciences, Isfahan, Iran; 2 Infectious Diseases and Tropical Medicine Research Center, Isfahan University of Medical Sciences, Isfahan, Iran

**Keywords:** *staphylococcus aureus*, methicillin-resistant *staphylococcus aureus*, RAPD methods, molecular typing, Iran

## Abstract

The aim of this study was to apply RAPD technique to analyze the genetic variability among the Iranian CA-MRSA isolates.

The RAPD amplification was implemented on 25 strains isolated from the anterior nares of 410 healthy children using four randomly selected oligonucleotide primers from the stocks available in our laboratory, including the primers 1254, GE6, OLP6 and OLP13 from our stock. The amplified PCR products were detected on a 1.5% agarose gel and subjected to further analysis to establish the band profiles and genetic relationships using the Gel Compar® program.

The Iranian CA-MRSA isolates produced distinct RAPD patterns which varied based on the primer used, however, the primer 1254 revealed highly polymorphic patterns consisting 5 discernable RAPD types (RT), “RT1” (12, 48%), “RT2” (8, 32%), “RT3” (3, 12%), and “RT4 and RT5”, (a single RAPD type each, 4%). Phylogenetic analysis based on RAPD profiles divided most of the CA-MRSA isolates into 2 distinct but related RAPD clusters, a small group and two single unrelated RAPD types.

This study shows that the simple and cost-effective but rather difficult to optimize RAPD fingerprinting could be used to evaluate genetic and epidemiological relationships of CA-MRSA isolates on condition that the patterns are obtained from carefully optimized laboratory tests.

## 1. Introduction

Methicillin resistant *S. aureus* (MRSA) spread is no longer limited to the hospitals, but has emerged in communities to infect an increasing number of people and in particular children (Green et al., 2012). Nasal carriage of community associated-methicillin resistant *S. aureus* (CA-MRSA) is a major source of endogenous infection as well as of human to human transmission (David & Daum, 2010). Accurate and rapid typing of *S. aureus* is crucial to the control of infectious organisms and numerous methods have been described elsewhere (Strandén, Frei, & Widmer, 2003; Mostafa, 2013).

Molecular techniques have been extensively developed for the last 30 years to study the genetic diversity of *S. aureus* strains and in particular MRSA strains (Mehndiratta & Bhalla, 2012; Vivoni & Moreira, 2005). These techniques have been validated in order to distinguish *S. aureus* strains for local epidemiologic or outbreak investigation purposes. The molecular approaches such as PCR (Rep-PCR), using primers that target repetitive extragenic palindromic DNA regions (Vecchio et al., 1995), multilocus enzyme electrophoresis (MLEE), a technique that analyzes the electrophoretic mobility of housekeeping enzymes (Boerlin, 1997), restriction fragment length polymorphism (RFLP) and DNA sequence analysis of the coagulase gene isolates (Hookey, Richardson, & Cookson, 1998), the repetitive extragenic palindromic have been applied in order to identify and investigate polymorphism within *S. aureus* strains. Pulsed-field gel electrophoresis (PFGE) and multilocus sequence typing (MLST) are currently considered the “gold standard” method for epidemiological investigation of MRSA; however, the feasibility of these methods in countries with limited financial and technical resources is a matter of concern (Prevost, Jaulhac, & Piemont, 1992; Murchan et al., 2003; Enright et al., 2000).

The aim of this study was to assess the genetic relationship of nasal carriage CA-MRSA isolates from healthy children in Iran by a more affordable, cost-effective, efficient, quick and easy to assay molecular technique, i.e., RAPD-PCR.

It is noted that the use of this technique has previously been reported in other countries including developing countries and Iran, as suitable for this purpose (Onasanya et al., 2003; Othman, 2010; Fitzgerald et al., 1997; Nikbakht et al., 2008).

## 2. Materials & Methods

### 2.1 Isolation and Identification of MRSA

A total of 25 CA-MRSA isolates from anterior nares of 410 healthy preschool children between 2 to 6 years of age without any known risk factors were investigated by the current study. All MRSA isolates were initially identified by conventional microbiological assay and further characterized by PCR amplification of Eap-encoding (*eap*) and mecA-encoding (*mecA*) genes as species and MRSA specific markers (Hussain et al., 2008; Jonas et al., 2002).

### 2.2 RAPD Analysis

Total genomic DNA was obtained using the method of Pitcher as previously described with minor modification for large-scale DNA extractions (Pitcher, Saunders, & Owen, 1989; Shojaei et al., 2000). RAPD-PCR assay was carried out according to procedure outlined by Burucoa, Lhomme, & Fauchere, 1999.

In brief, PCR amplification was performed in a 25 µl total volume containing 1U Taq DNA polymerase, 2.5 µl of 10xPCR buffer, 2µl of the randomly selected primers from our library, i.e., the primers GE6 (5’-CCC GTC AGC A-3’), 1254 (5’-CCG CAG CCA A-3’), OLP6 (5’- GAGGGAAGAG-3’), OLP13 (5’- ACCGCCTGCT-3’) and 50 ng of DNA template. PCR cycling parameters were as follows: an initial hot start at 95ºC followed by4 cycles each consisted of 94ºC for 1 minute, 30ºC for 1 minute, and 72ºC for 2 minutes, followed by 36 cycles of 94ºC for 1 minute, 36ºC for 1 minute, and 72ºC for 2 minutes and a final extension step of 72ºC for 5 minutes. A negative control of the same reaction mixture without DNA and a positive control, containing chromosomal DNA of the reference strain MRSA ATCC 33591 were included in each run. Each isolate was tested under the same conditions at least twice with the selected oligonucleotides. Amplified PCR fragments were subjected to electrophoresis in a 1.5% agarose gel at a constant voltage of 4V/cm and scanned by the Gel Documentation Systems. The software DNA FRAG version 3.03 (Nash, 1991) was used to estimated DNA fragment sizes in the RAPD profiles.

The RAPD profiles were defined based on the number and position of the major bands according to the Dice formula and similarity coefficients for each pair of lanes and a dendrogram generated based on the unweighted pair-group method with averages (UPGMA) by means of the GelCompar 3.1 software (Applied Maths, Kortrijk, Belgium) to estimate the relationships between the isolates.

## 3. Results

The RAPD analysis with DNA from the CA-MRSA isolates, and the primers OLP6, OLP13 and GE6 was found to yield less informative banding patterns.

**Figure 1:**
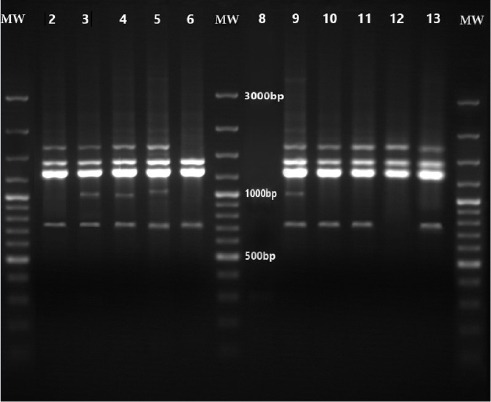
RAPD-PCR assay for CA-MRSA Lanes: 1, 7 and 14: MW, 100-bp DNA ladder marker, Lanes: 2, 10, 11, 13: RAPD type 1, Lanes: 3, 4: RAPD type 2, Lane 5: RAPD type 4, Lane 6: RAPD type 5, Lane 13: RAPD type 3

**Figure 2:**
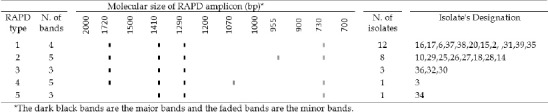
Normalized graph showing the details of RAPD profiles of Iranian nasal carriage CA-MRSA

However, primers 1254 yielded the best RAPD patterns with regard to number, distribution and intensity of the bands. Consequently, it was selected to be used with all strains for typing analysis. RAPD analysis with the chosen primer generated 5 different patterns. These genotypes assigned to RAPD types 1 to 5 (Figure 1). The RAPD profiles consisted of three to five amplicons ranging from 730 to 1720 base pairs in length. Apart from one isolate, i.e., strain ST34, all other isolates generated some conserved bands that could be detected in the patterns from all isolates, that is, the fragments of 1290, 1410 and 1720 base pairs. The isolate ST34 lacked a 1720 bp fragment (Figure 2). According to our results, RAPD types 1 with 12 isolates (48%) was the most frequently encountered, followed by RAPD type 2 with 8 isolates (32%) as the second, RAPD types 3 (3 isolates) as the third then RAPD types 4 and 5 (each contains 1 isolates), as the fourth and the fifth RAPD types (Figure 2).

**Figure 3:**
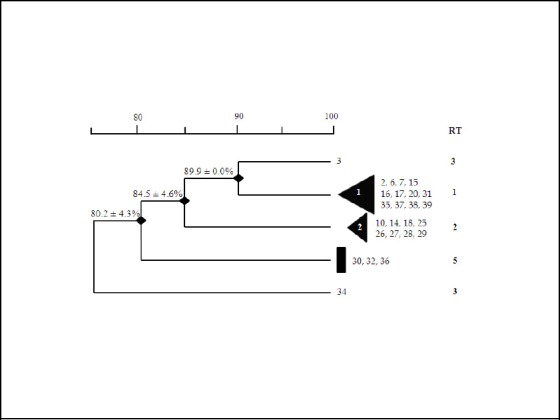
Dendrogram of the nasal carriage CA-MRSA RAPD-PCR DNA fingerprints with the primer 1254; Similarity coefficients are included in the top bar

Dendrogram analysis of RAPD-PCR amplification patterns of the Iranian isolates of CA-MRSA resulted in formation of 2 distinct but related RAPD clusters. RAPD cluster 1 had the highest similarity (89.9%) with RAPD type 4. The remaining 3 isolates occurred in a small group that showed a similarity of 84.5± 4.6% with the other two clusters and RAPD type 4. The RT 5 also formed a rare single line of descent which was distantly related to other isolates with a similarity of 80.2 ± 4.3 % (Figure 3).

## 4. Discussion

Molecular typing methods have been evaluated not only for their ability to discriminate among strains for epidemiologic purposes but also for their potential as taxonomic value. Previous studies have shown that *S. aureus* is a polymorphic species and has a clonal population structure (Fitzgerald et al., 2001; Feil et al., 2003). Several molecular methods have been used for epidemiological surveillance of MRSA isolates in order to track the distribution, infection source and transmission routes (Tenover et al., 1994; van Belkum et al., 1995; Hookey, Richardson, & Cookson, 1998; Vecchio et al., 1995; Boerlin, 1997). The typing techniques that are most commonly used today for typing of MRSA strains are multilocus sequence typing (MLST) (Enright et al., 2000), pulsed-field gel electrophoresis (PFGE) (Prevost, Jaulhac, & Piemont, 1992; Murchan et al., 2003), spa typing (Frenay et al., 1996), and SCC mec typing (Oliveira & de Lencastre, 2002; Ito et al., 2001).

Most of these methods suffer from the disadvantages that require high expertise, complicated laboratory settings and complex procedures to clearly differentiate the various MRSA. In our study we intended to find the molecular variation of Iranian isolates of CA-MRSA by utilizing a rather simple and cost-effective molecular typing method, namely, random amplified polymorphic DNA (RAPD) analysis (Welsh & McClelland, 1990; Reinoso et al., 2004). Compared with other typing methods for *S.aureus* strains such as PFGE (Prevost, Jaulhac, & Piemont, 1992), this procedure generates greater polymorphism, is technically friendly and faster, and requires no radioactive materials (Welsh & McClelland, 1990; Reinoso et al., 2004; Linton et al., 1995).

Our results indicated that RAPD fingerprinting can classify isolates of MRSA into clusters through which the relationship of strains can be evaluated**. Previous studies has documented that the polymorphism generated by RAPD is almost the same as that yielded by RFLP (Linton et al., 1995). However, RAPD is almost incomparable in terms of simplicity, fastness and low-cost conditions with most other molecular typing methods (Burucoa, Lhomme, & Fauchere, 1999; Welsh & McClelland, 1990). In addition, a smaller volume of total DNA is required than for methods such as hybridization based methods (Welsh & McClelland, 1990; Linton et al., 1994). This is of obvious significance in terms of cost analysis as, in our experience; DNA isolation from *staphylococcus aureus* is almost unlikely without using a rather expensive enzyme, that is, lysostaphin (Enright et al., 2000). Although RAPD is simple and useful for epidemiological analysis, optimization of the PCR conditions is very important for reliability and reproducibility of the polymorphic patterns (Burucoa, Lhomme, & Fauchere, 1999). For instance in our experiment it was highly crucial to optimize the amount of DNA in each RAPD assay (50 ng of DNA) to ensure that non-specific bands were not present.

In fact our rather long involvement in utilization of RAPD as a favorite typing techniques for developing countries has shown that, even with rigid precaution and standardization, it is rather difficult to obtain reproducible patterns with RAPD assay and as far as our experiences concern it is of high practical significance to carry out the RAPD experiment in duplicate for all isolates in order to acquire the true profile differences for the studied strains.

In our investigation the RAPD application in CA-MRSA genotype analysis based on primer 1254, resulted in 5 RAPD types for 25 isolates. We classified CA-MRSA strains as either cluster (containing >3 members), small group (2 or 3 members) or unique, i.e., single isolates. Of 25 CA-MRSA isolates, 20 (80%) grouped into two clusters, i.e., 1 &2, and 3 isolates (12%) classified into a small group, and 2 isolates (8%) had a unique RAPD profile. The RAPD types 1 and 2 that encompassed 80% of all isolates prevailed. From this finding it can be inferred that RT 1 and RT2 are the major prevalent strains circulating in our community. Whether these strains have a particular feature that facilitates their colonization and adaptation capacity within Iranian children is a question that remains to be answered by further studies including more advance molecular typing assay such as MLST.

## 5. Conclusion

Taking into account the fact that advances in the development of more accurate and reproducible molecular methods have offered new typing choices for *S.aureus* strains, such as PFGE, MLST and spa typing, our data indicated that a simple, rapid and cost-efficient but rather difficult to optimize RAPD-PCR can be successfully utilized to find out the distributional and epidemiological relationship of CA-MRSA isolates provided that the technique is implemented under careful reproducibility condition and in particular duplication of PCR runs to obtain valid results.
